# Porcine Reproductive and Respiratory Syndrome Virus Adapts Antiviral Innate Immunity via Manipulating MALT1

**DOI:** 10.1128/mbio.00664-22

**Published:** 2022-04-25

**Authors:** Han Gu, Suya Zheng, Guangwei Han, Haotian Yang, Zhuofan Deng, Zehui Liu, Fang He

**Affiliations:** a Institute of Preventive Veterinary Medicine, College of Animal Sciences, Zhejiang University, Hangzhou, China; b Zhejiang Provincial Key Laboratory of Preventive Veterinary Medicine, Hangzhou, China; College of Veterinary Medicine, Cornell University

**Keywords:** MALT1, MCPIP1, N4BP1, PRRSV, RNase, innate immunity, nsp

## Abstract

To fulfill virus replication and persistent infection in hosts, viruses have to find ways to compromise innate immunity, including timely impedance on antiviral RNases and inflammatory responses. Porcine reproductive and respiratory syndrome virus (PRRSV) is a major swine pathogen causing immune suppression. MALT1 is a central immune regulator in both innate and adaptive immunity. In this study, MALT1 was confirmed to be induced rapidly upon PRRSV infection and mediate the degradation of two anti-PRRSV RNases, MCPIP1 and N4BP1, relying on its proteolytic activity, consequently facilitating PRRSV replication. Multiple PRRSV nsps, including nsp11, nsp7β, and nsp4, contributed to MALT1 elicitation. Interestingly, the elevated expression of MALT1 began to decrease once intracellular viral expression reached a high enough level. Higher infection dose brought earlier MALT1 inflection. Further, PRRSV nsp6 mediated significant MALT1 degradation via ubiquitination-proteasome pathway. Downregulation of MALT1 suppressed NF-κB signals, leading to the decrease in proinflammatory cytokine expression. In conclusion, MALT1 expression was manipulated by PRRSV in an elaborate manner to antagonize precisely the antiviral effects of host RNases without excessive and continuous activation of inflammatory responses. These findings throw light on the machinery of PRRSV to build homeostasis in infected immune system for viral settlement.

## INTRODUCTION

Porcine reproductive and respiratory syndrome virus (PRRSV) is widely prevalent in pigs, causing huge economic losses to the global swine industry (1). PRRSV, an enveloped, single-stranded, positive-sense RNA virus, is a member of the genus *Porartevirus*, family *Arteriviridae*, and order *Nidovirales* (2). Its genome is about 15.4 kb in length and contains at least 11 open reading frames (ORFs), encoding 8 structural proteins and at least 16 nonstructural proteins (nsps): nsp1α, nsp1β, nsp2TF, nsp2N, nsp2-6, nsp7α, nsp7β, and nsp8 to -12 (3). PRRSV is an immunosuppressive pathogen, inducing poor innate immune responses, especially the suppression of type I interferons (IFNs) (4, 5). However, the mechanism for PRRSV-mediated immune suppression is not entirely clear. Because of the typical immune suppression, PRRSV could be a representative of immunosuppressive pathogens, provide enlightening clues for other viruses, like human immunodeficiency virus (HIV). PRRSV usually goes through acute and persistent infection stages, and PRRSV-modulated immune state favors its chronic persistent infection. In the acute infection stage, PRRSV replicates mainly in respiratory macrophages and dendritic cells (DC), causing viremia lasting for several weeks. During persistent infection, virus replication is limited to lymphoid organs like tonsils and lymph nodes. Although pigs no longer exhibit overt symptoms, viruses maintain replication for several months, keeping viruses shedding and spreading in the herd. Hence, pigs may experience a lifelong infection of PRRSV in a typical swine production setting (1). Although commercial attenuated vaccines are widely used currently, PRRSV has not been effectively controlled yet due to occurrences of viral shedding, recombination with field strains, virulence reversion, and interference in other swine vaccines (6–9).

Monocyte chemotactic protein-induced protein (MCPIP1), or Zc3h12a or Regnase-1, belongs to the Zc3h12a-like NYN domain subfamily of endoribonucleases. All members of the Zc3h12a-like RNase family have a similar PilT N terminus (PIN)-like domain with RNase activity, involved in antiviral immunity (10). MCPIP1 is a broad-spectrum antiviral protein, suppressing infection of HIV (11) and many other viruses. Recently, MCPIP1 was confirmed to also inhibit PRRSV replication (12). NEDD4-binding protein 1 (N4BP1), another Zc3h12a-like RNase, also inhibits HIV-1 replication via its PIN-like RNase structure (13). However, MCPIP1 and N4BP1 are cleaved and inactivated by mucosa-associated lymphoid tissue lymphoma translocation protein 1 (MALT1) in activated T cells, reactivating latent HIV-1 (11, 13, 14).

MALT1, also known as paracaspase-1 (PCASP1), is an intracellular signaling protein that is widely expressed in host cells. It contains an N-terminal death domain, two Ig-like domains, and a C-terminal caspase-like domain (15). MALT1 often binds to Bcl10 and CARMA1 through its Ig-like domains, assembling the canonical CARMA1-Bcl10-MALT1 (CBM) complex (15). On the one hand, as a scaffold protein in this supramolecular complex, MALT1 recruits the E3 ligase TRAF6, triggering a cascade of downstream reactions and, as a result, causing activation of the NF-κB signaling pathway (16, 17). On the other hand, as a protease, MALT1 degrades a range of specific substrates via its caspase-like domain. MALT1 cleaves Bcl-10, deubiquitinases A20 and CYLD, and NF-κB subunit RelB, thereby modulating NF-κB and JNK signaling (18–21). RNA-binding proteins, such as MCPIP1, Roquin-1, and Roquin-2, are also substrates of MALT1. By targeting these endoribonucleases, MALT1 improves the stability of downstream mRNAs of a great number of proinflammatory cytokines, regulating innate immunity at the posttranscriptional level (22). In addition, MALT1 proteolytic activity is also essential for the differentiation of lymphocytes including Tregs, marginal-zone B cells, and B1 B cells (22). Together, MALT1 is a crucial signaling component, as well as a protease, regulating innate and adaptive immunity, especially activation of NF-κB and inflammatory responses.

MALT1 has been confirmed to be involved in the pathogenicity of several viruses. MALT1 facilitates the reactivation of latent HIV-1 proviruses in CD4^+^ T cells by cleaving N4BP1 (13). MALT1-mediated early inflammatory responses and T cell activation are of great significance to inhibit rabies virus (23), whereas inhibition of MALT1 can alleviate the neuroinflammation caused by rabies virus infection and prolong the survival time of infected mice (24). MALT1 mediates the production of MMP-9 in alveolar macrophages, which is involved in influenza pathogenesis and exacerbates the severity of influenza (25). Hence, MALT1 plays a double-edged role in virus pathogenesis and host immunity, but the function in PRRSV is unclear.

In this study, the regulatory relationship among PRRSV, innate antiviral defense, and MALT1 was explored. First, the suppressive function of N4BP1 and MCPIP1 on PRRSV replication was identified. Subsequently, the degradation on both RNases by MALT1 were confirmed, and the effects on virus replication were explored. Moreover, MALT1 expression profile at different PRRSV infection levels was evaluated, and the vital PRRSV nsps with significant regulatory effects on MALT1 were screened, revealing the precise control of expression of MALT1 by PRRSV. In addition, the effects of PRRSV regulating MALT1 inflammatory responses were explored. This study revealed a new mechanism of PRRSV against the innate immune defense by manipulating MALT1 to antagonize anti-PRRSV RNases and alleviate inflammatory responses.

## RESULTS

### N4BP1 and MCPIP1 inhibit PRRSV replication, relying on their RNase activity.

**FIG 1:**
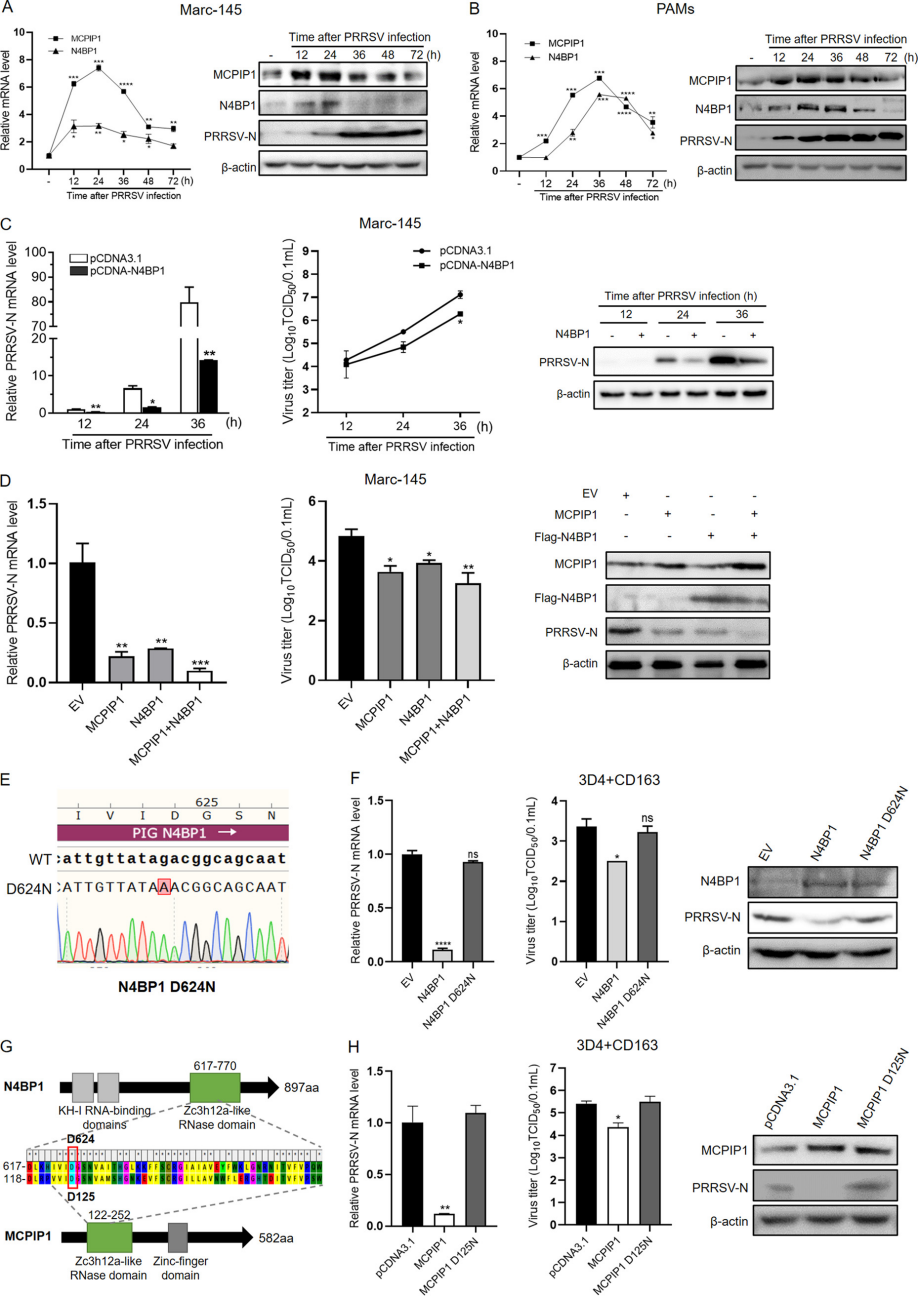
N4BP1 and MCPIP1 inhibit PRRSV replication via their RNase activities. (A and B) Marc-145 cells (A) or PAMs (B) were infected with PRRSV HuN4 (MOI of 1) for the indicated time. RNA and protein were extracted from cell lysates to detect N4BP1 and MCPIP1 expression in qRT-PCR and Western blotting, respectively. (C) Marc-145 cells were transfected with pCDNA-N4BP1 or empty plasmid for 24 h and then infected with PRRSV. At 12, 24, and 36 hpi, PRRSV-N mRNA levels (left), virus titers of culture supernatants (middle), and N protein expression (right) were determined. (D) Marc-145 cells were transfected with MCPIP1 or N4BP1 individually or the combination of the two plasmids for 24 h. Cells were then infected with PRRSV for 24 h. PRRSV-N mRNA levels (left), virus titers (middle), and N protein expression (right) were detected. (E) The construction of pCDNA3.1-N4BP1 D624N. (F) 3D4+CD163 cells were transfected with N4BP1, D624N, or empty vector for 24 h. Cells were then infected with PRRSV for 24 h. PRRSV-N mRNA levels (left), virus titers (middle), and N protein expression (right) were detected. (G) Amino acid sequence alignment of RNase domain of pig N4BP1 and MCPIP1. D624 of porcine N4BP1 and D125 of porcine MCPIP1 are homologous sites. (H) 3D4+CD163 cells were transfected with MCPIP1, D125N, or empty vector for 24 h. Cells were then infected with PRRSV for 24 h. PRRSV-N mRNA levels (left), virus titers (middle), and N protein expression (right) were detected.

Expression levels of the two RNases (N4BP1 and MCPIP1) upon PRRSV infection were detected in quantitative reverse transcription-PCR (qRT-PCR) and Western blot analysis. The results showed that PRRSV infection induced RNase expression rapidly in Marc-145 cells and porcine alveolar macrophages (PAMs), especially before 36 h postinfection (Fig. 1A and B). However, the expression levels decreased during the late stage of infection. These results indicated that host RNases N4BP1 and MCPIP1 were rapidly induced by PRRSV infection.

To assess the impact of N4BP1 on PRRSV replication, Marc-145 cells were transfected with N4BP1 expression plasmids or empty vector control and then infected with PRRSV. Virus replication levels were detected over a 36-h infection course in qRT-PCR and Western blot analysis with infected culture supernatants. As shown in Fig. 1C, the expression of PRRSV N protein was significantly inhibited in both mRNA and protein levels after overexpressing N4BP1 compared to the vector control. In addition, the virus titers in culture supernatants also were reduced after N4BP1 overexpression. The suppressive effects of MCPIP1 on PRRSV infection were demonstrated in our recent study (12). In addition, to evaluate whether N4BP1 has a synergistic effect with MCPIP1 in inhibiting PRRSV, Marc-145 cells were transfected with MCPIP1 or N4BP1 plasmids, respectively, or cotransfected with the two plasmids. The results of qRT-PCR, virus titers, and Western blot analysis showed that cotransfection of MCPIP1 and N4BP1 has a more significant suppressive effect on PRRSV replication than either single transfection (Fig. 1D). These results demonstrated the inhibitory activity of N4BP1 and MCPIP1 against PRRSV replication and the cooperative antiviral effects of these two RNases.

To further investigate whether N4BP1 and MCPIP1 antagonize PRRSV infection via their RNase domains, mutants of pig-derived N4BP1 and MCPIP1 with deficiency of RNase activity were constructed. Asp623 of human N4BP1 has been proven as the essential site for its catalytic center, and N4BP1 D623N mutant fully abrogated its RNase activity on HIV-1 mRNA (13). According to the amino acid sequence alignment of RNase domain of pig and human N4BP1, their RNase domains are highly conserved, and D624 of porcine N4BP1 is consistent with D623 of human N4BP1. Therefore, a point mutation of D624N of porcine N4BP1 was constructed (Fig. 1E). On this basis, the antiviral effects against PRRSV of wild-type (WT) N4BP1 and D624N mutant were compared. The results of PRRSV N mRNA and protein levels, along with virus titers, showed that D624N mutant was unable to suppress PRRSV replication in 3D4+CD163 cells at 24 hpi (Fig. 1F). A similar mutation was induced to porcine MCPIP1. The RNase domain structure and primary amino acid sequence of N4BP1 are similar to those of MCPIP1 (13). As indicated in the comparison of RNase domain amino acid sequences between porcine N4BP1 and MCPIP1, D624 of porcine N4BP1 and D125 of porcine MCPIP1 are homologous sites (Fig. 1G). Hence, MCPIP1 D125N mutant was constructed and assessed for the inhibitory effects on PRRSV. The results showed that wild-type MCPIP1 significantly suppressed PRRSV replication, whereas D125N mutation abolished the anti-PRRSV activity in MCPIP1 (Fig. 1H). Taken together, these results verified N4BP1 and MCPIP1, the two host RNases, suppressed PRRSV replication via their RNase activity.

### MALT1 protease degrades N4BP1 and MCPIP1, facilitating PRRSV replication.

**FIG 2:**
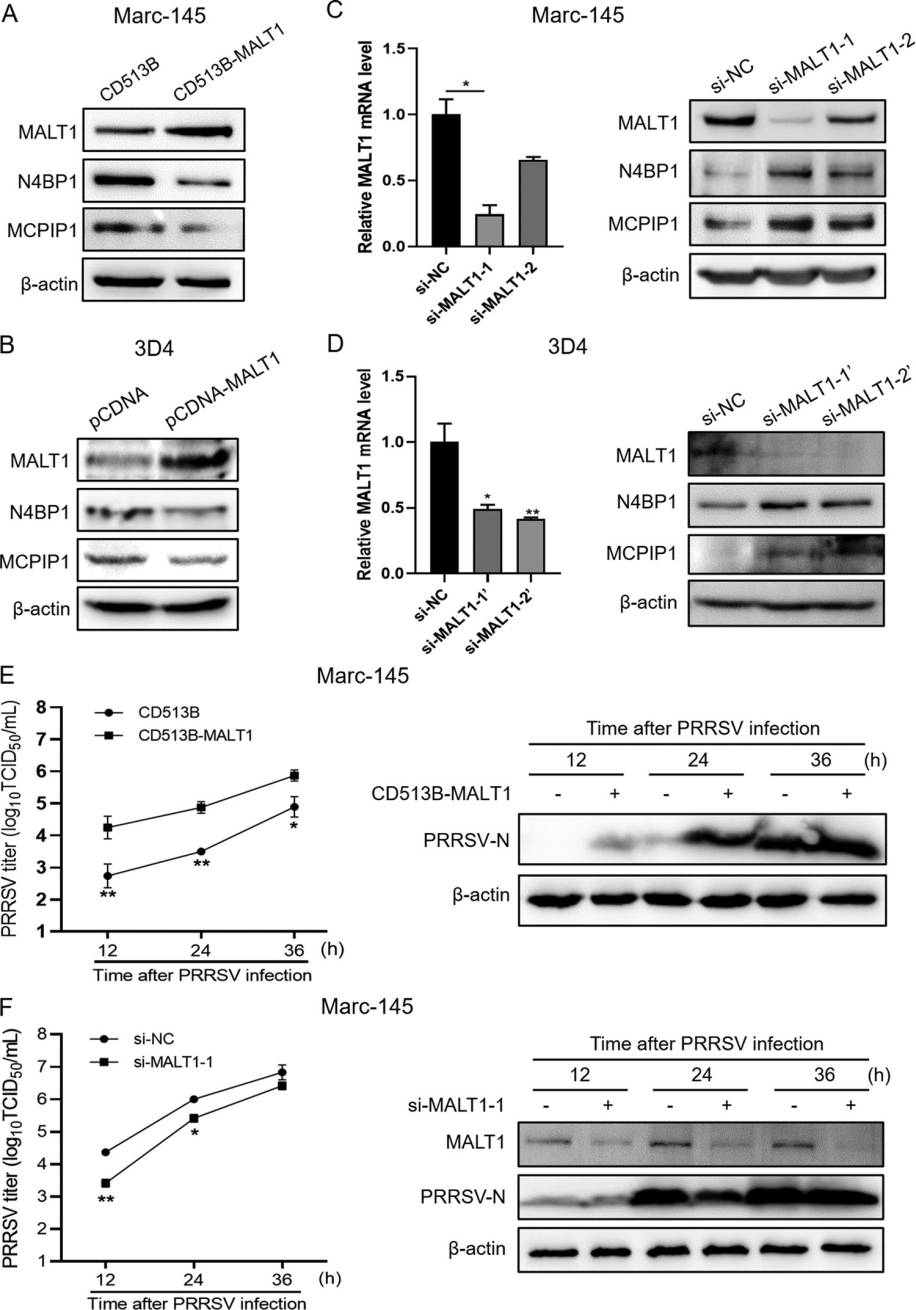
MALT1 antagonizes protein expression of N4BP1 and MCPIP1 and facilitates PRRSV replication. (A and B) Marc-145 (A) and 3D4 (B) cells were transfected with MALT1 expression plasmids or empty vector. MALT1, N4BP1, and MCPIP1 protein expression was detected in Western blot analysis. (C and D) Marc-145 (C) and 3D4 (D) cells were transfected with two small interfering RNAs (siRNAs) targeting MALT1 mRNA or si-NC for 48 h. MALT1, N4BP1, and MCPIP1 protein expression was detected in Western blot analysis. (E) Marc-145 cells were transfected with CD513B-MALT1 or empty plasmid for 24 h and then infected with PRRSV for the indicated time. Virus titers (left) and PRRSV-N protein expression (right) were detected. (F) Marc-145 cells were transfected with si-MALT1-1 or si-NC for 24 h and then infected with PRRSV. Virus titers (left) and PRRSV-N protein expression (right) were detected at the indicated time postinfection.

Previous studies confirmed that in T cells, MALT1 protease cleaves N4BP1 and MCPIP1 (13, 22). Here, to verify the effects of MALT1 on the expression of the two RNases in Marc-145 and 3D4 cells, MALT1 overexpression and knockdown assays were performed. As shown in Fig. 2A and B, monkey- or pig-derived MALT1 was overexpressed in Marc-145 or 3D4 cells, respectively, causing a significant decrease in N4BP1 and MCPIP1 protein expression. Consistent with this, knockdown of MALT1 by siRNA in Marc-145 and 3D4 cells markedly upregulated the protein levels of the two RNases (Fig. 2C and D). Furthermore, PRRSV replication levels were determined in MALT1 overexpressed or downregulated Marc-145 cells. The results showed that MALT1 overexpression significantly upregulated virus titers in the culture supernatant and PRRSV-N protein levels in infected cells during a 36-h infection course (Fig. 2E). On the other hand, knockdown of MALT1 by short interfering RNA (siRNA) dramatically downregulated viral replication levels (Fig. 2F).

**FIG 3:**
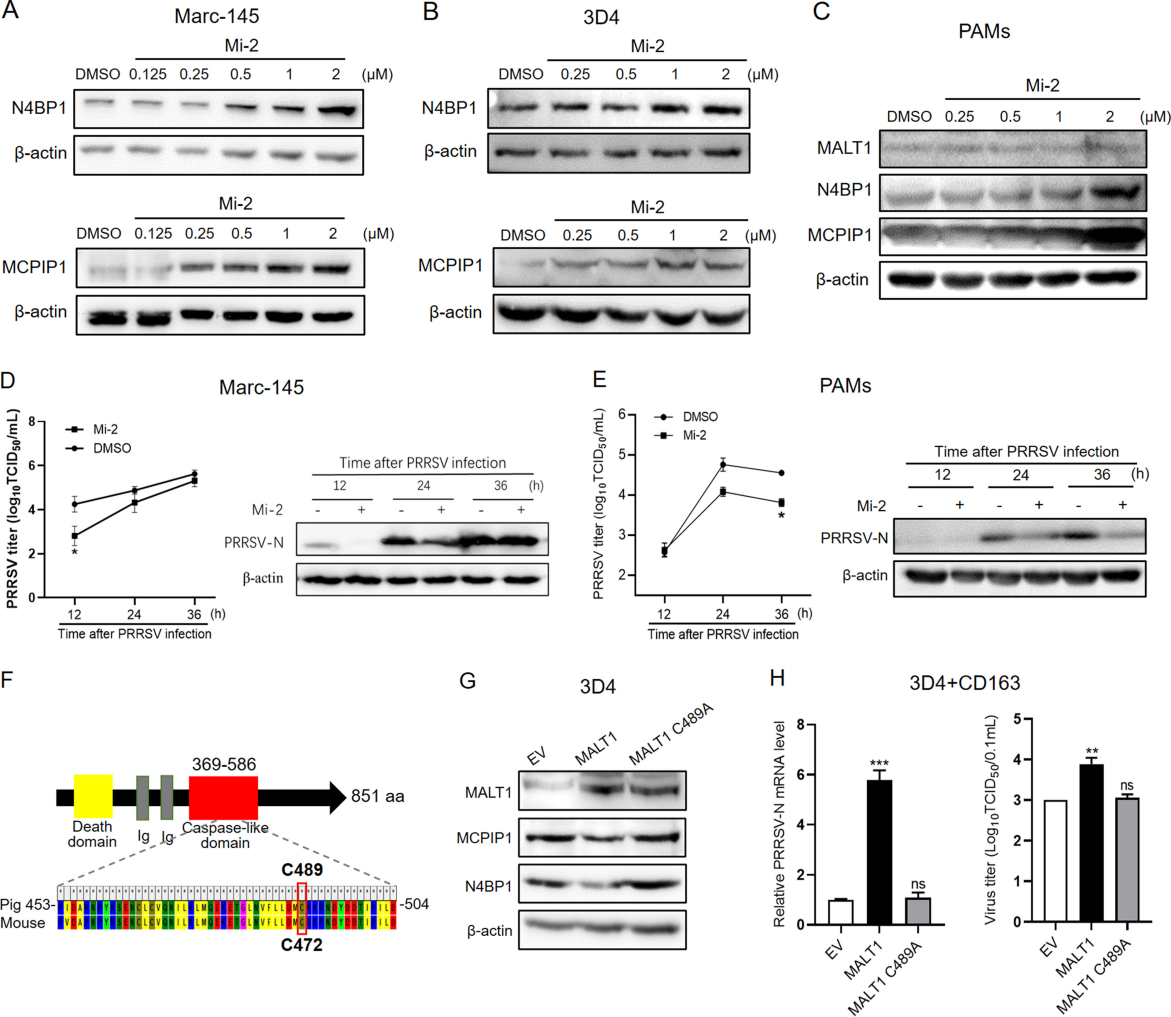
MALT1-mediated degradation of RNases relies on its proteolytic activity. (A to C) Marc-145 cells (A), 3D4 cells (B), and PAMs (C) were incubated with different concentrations of Mi-2 (0 to 2 μM) for 24 h. N4BP1 and MCPIP1 protein expression was detected in Western blot analysis. (D and E) Marc-145 cells (D) and PAMs (E) were incubated with 1 μM Mi-2 or DMSO for 24 h and then infected with PRRSV for 12, 24, or 36 h. Virus titers (left) and PRRSV-N protein levels (right) were detected. (F) The domain architecture of porcine MALT1 and amino acid sequence alignment of part of caspase-like domain of pig and mouse MALT1. C489 of porcine MALT1 and C472 of mouse MALT1 are homologous sites. (G) 3D4 cells were transfected with MALT1, C489A, or empty vector for 24 h. MALT1, MCPIP1, and N4BP1 protein expression was detected in Western blot analysis. (H) 3D4+CD163 cells were transfected with MALT1, C489A, or empty vector for 24 h. Cells were then infected with PRRSV for 24 h. PRRSV-N mRNA levels (left) and virus titers (right) were detected.

To further clarify whether proteolytic activity of MALT1 functions in antagonizing RNase expression, Mi-2, a specific MALT1 inhibitor, was used. Mi-2 can covalently bind to the caspase-like domain of MALT1 and suppress its protease function (18). The results showed that different concentrations of Mi-2 (0 to 2 μM) markedly upregulated N4BP1 and MCPIP1 protein expression in a dose-dependent manner in Marc-145 cells (Fig. 3A) as well as in 3D4 cells (Fig. 3B). Similarly, protein levels of the two RNases increased upon Mi-2 treatment in PAMs in a dose-dependent manner (Fig. 3C). Importantly, Mi-2 treatment significantly downregulated PRRSV replication levels in Marc-145 cells and PAMs during a 36-h infection course (Fig. 3D and E).

C472 of mouse MALT1 in its caspase-like domain has been demonstrated as the vital site to maintain its proteolytic activity (22). In porcine MALT1, C489 is homologous to C472 of mouse-derived protein (Fig. 3F). Thus, C489A mutant of pig MALT1 was constructed and used to transfect 3D4 cells. The results of Western blot analysis showed that N4BP1 and MCPIP1 protein expression was significantly downregulated after MALT1 overexpression, whereas C489A had no regulatory effects on their expression (Fig. 3G). Moreover, quantitative PCR (qPCR) and virus titer detection revealed that MALT1 overexpression notably upregulated PRRSV replication levels in 3D4+CD163 cells compared with the empty vector group, whereas no marked impact on viral replication induced by C489A was detected (Fig. 3H).

These results indicated that MALT1 degraded and downregulated N4BP1 and MCPIP1 relying on its proteolytic activity, thus facilitating PRRSV replication.

### MALT1 expression is elevated upon PRRSV infection.

**FIG 4:**
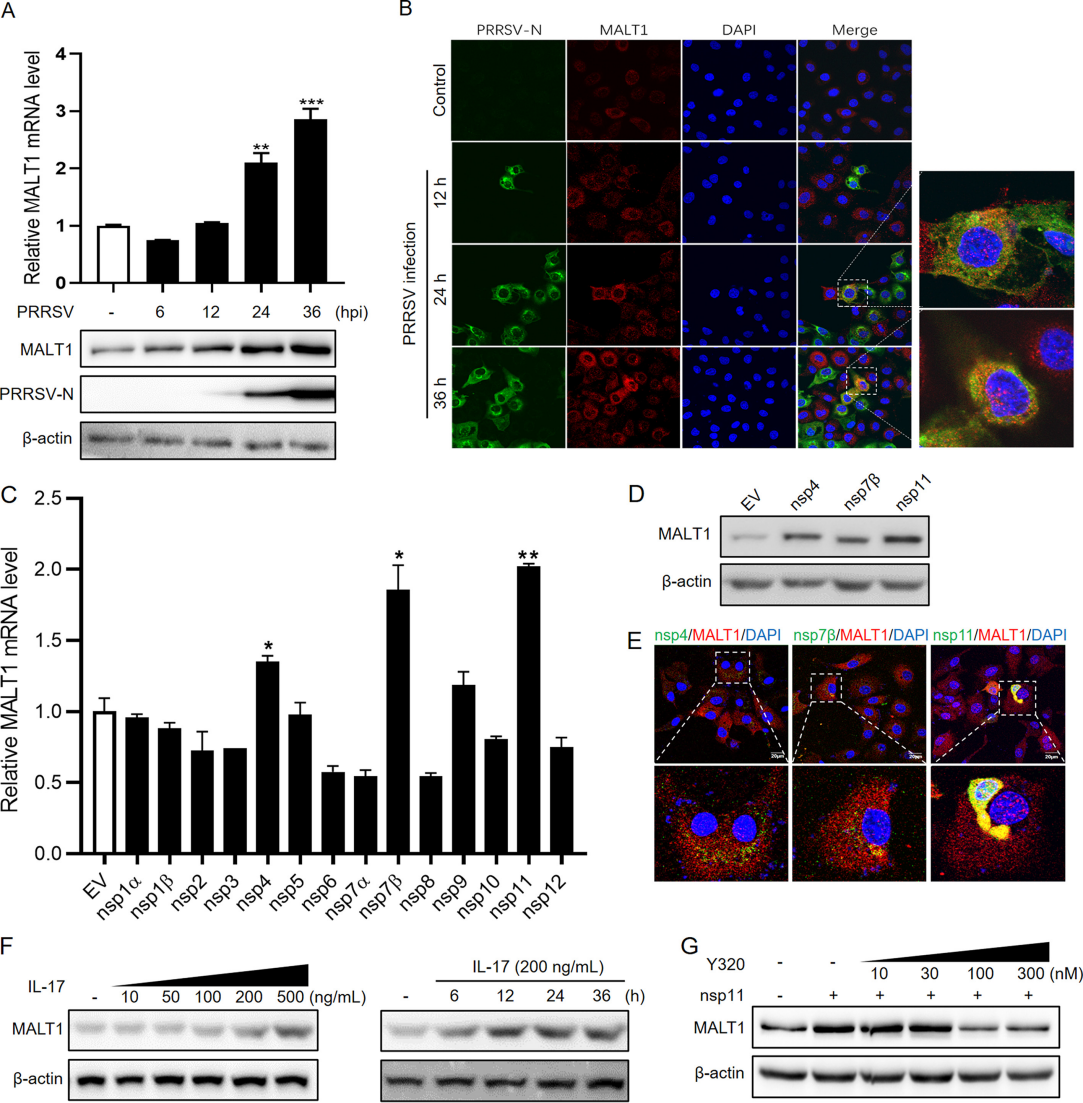
MALT1 expression is elevated upon PRRSV infection. (A and B) Marc-145 cells were infected with PRRSV HuN4 (MOI, 1) for the indicated time periods. (A) MALT1 mRNA and protein expression was detected in qRT-PCR and Western blot, respectively. (B) PRRSV N protein and MALT1 were immunostained with anti-PRRSV N MAb VH13 (green) and anti-MALT1 pAb (red), respectively. Nuclei were labeled with DAPI (blue). (C) Marc-145 cells were transfected with plasmids expressing different nsps or pCDNA3.1 empty vector (EV). mRNA levels of MALT1 were detected by qRT-PCR 24 h after transfection. (D) Marc-145 cells were transfected with nsp4, -7β, or -11 plasmids or EV. MALT1 protein levels were detected in Western blot analysis. (E) Marc-145 cells were cotransfected with His-nsp and MALT1 plasmids. nsp and MALT1 signaling was visualized using anti-His MAb (green) and anti-MALT1 MAb (red), respectively. (F) Marc-145 cells were incubated with different concentrations of IL-17 (10 to 500 ng/mL) for 12 h (left) or with 200 ng/mL of IL-17 for different time periods (right). MALT1 levels were detected in Western blot analysis. (G) Marc-145 cells were incubated with IL-17 inhibitor Y320 at different concentrations (0 to 300 nM) for 24 h and subsequently transfected with pCDNA-nsp11 or empty vector. Culture supernatant was replaced with complete medium containing Y320 at indicated concentrations at 6 h posttransfection. Cells were collected 24 h after transfection for detection of MALT1 expression in Western blot analysis.

To evaluate the impact of PRRSV infection on MALT1 expression, mRNA and protein levels of MALT1 upon PRRSV infection in Marc-145 cells were first detected using qPCR and Western blot analysis, respectively. The results showed that over a 36-h infection course, MALT1 was remarkably upregulated both in gene and protein levels (Fig. 4A). Moreover, MALT1 expression was visualized by confocal microscopy. Fluorescence signals of PRRSV N and MALT1 protein were significantly increased after PRRSV infection (Fig. 4B). Notably, merged images indicated that PRRSV N protein was colocalized with MALT1, suggesting the interaction between MALT1 and PRRSV. These results demonstrated that MALT1 could be induced by PRRSV infection.

To investigate the viral factors that induce MALT1 expression, Marc-145 cells were transfected with various plasmids expressing PRRSV nsps. The results showed that MALT1 mRNA expression was upregulated upon transfection with nsp4, -7β, and -11 (Fig. 4C). Further, MALT1 protein was significantly induced after transfection with nsp4, -7β, or -11 compared with the empty vector group, especially with nsp11 (Fig. 4D). In addition, remarkable colocalization of nsp11 and MALT1 was observed in cells cotransfected with different nsps and MALT1 (Fig. 4E). PRRSV nsp11 has been confirmed to induce interleukin-17 (IL-17) expression, causing MCPIP1 downregulation (12). Thus, to investigate whether nsp11-induced IL-17 upregulates MALT1 expression, IL-17 recombinant protein was incubated with cells, and the results showed that IL-17 treatment markedly upregulated MALT1 protein level in dose- and time-dependent manners (Fig. 4F). On the contrary, IL-17 inhibitor Y320 dramatically suppressed nsp11-induced MALT1 expression (Fig. 4G). These results indicated that PRRSV nsp11 employed IL-17 to induce MALT1 expression.

### MALT1 is downregulated upon PRRSV infection aggravation.

**FIG 5:**
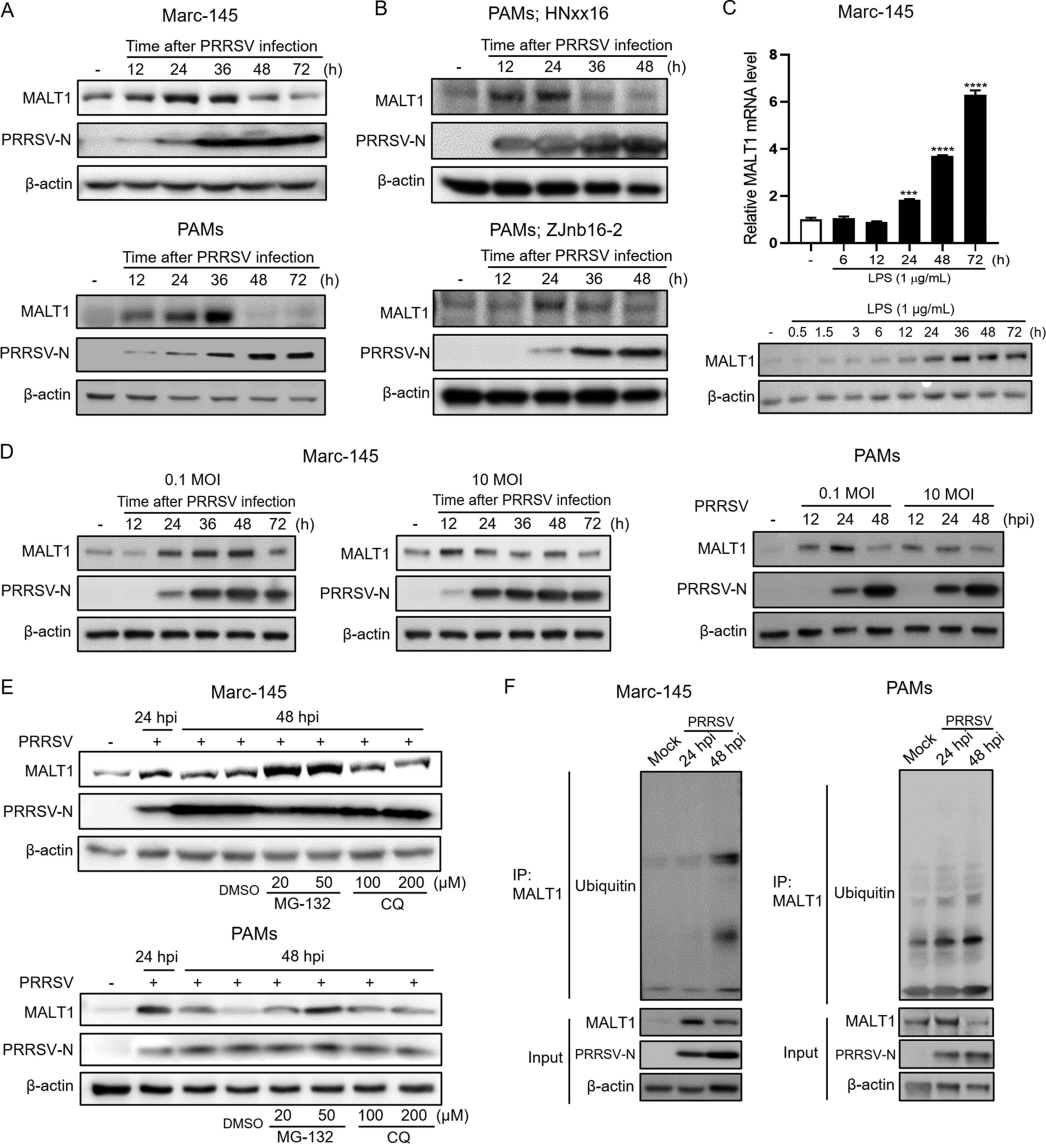
MALT1 is downregulated via ubiquitin-proteasome pathway upon PRRSV infection aggravation. (A) Marc-145 cells or PAMs were infected with PRRSV HuN4 (MOI, 1), and protein levels of MALT1 and PRRSV N were detected in Western blot analysis during a 72-h infection course. (B) PAMs were infected with PRRSV HNxx16 or ZJnb16-2 strain (MOI, 0.1), and MALT1 proteins were detected at the indicated time points. (C) Marc-145 cells were stimulated with 1 μg/mL LPS. MALT1 mRNA (top) and protein (bottom) levels were detected at the indicated time after LPS treatment. (D) Marc-145 cells and PAMs were infected at a PRRSV HuN4 MOI of 0.1 or 10, and MALT1 and PRRSV-N protein levels were determined at the indicated time points. (E) Marc-145 cells (top) and PAMs (bottom) were infected with HuN4 (MOI, 1) for the indicated time periods. MG-132 or chloroquine (CQ) at the indicated concentrations was added into the culture supernatants at 40 hpi. MALT1 levels were determined in Western blot analysis. (F) Marc-145 cells and PAMs were infected with HuN4 (MOI, 1), and cell lysates were collected at 24 and 48 hpi. Cell lysates were incubated with MALT1 antibodies and protein A+G agarose. Immunoprecipitates and whole-cell lysates were separated in SDS-PAGE and immunoblotted using anti-ubiquitin and anti-MALT1 antibodies.

Intriguingly, 36 h after PRRSV HuN4 infection, a significant decrease in MALT1 protein level was observed in both Marc-145 cells and PAMs (Fig. 5A). Similar trends of MALT1 expression also were observed in PAMs infected with other PRRSV strains, including low-pathogenicity strain HNxx16 and highly pathogenic strain ZJnb16-2 (6, 26) (Fig. 5B). MALT1 levels were upregulated before 24 hpi and started to decrease at 36 hpi. However, when stimulated with lipopolysaccharide (LPS), MALT1 mRNA and protein levels kept increasing without any decrease over the entire treatment of 72 h (Fig. 5C), indicating that generally PAMP stimulation evokes a continuous activation of MALT1 and the downregulation observed is uniquely operated by PRRSV. To further verify the correlation between severity of PRRSV infection and MALT1 expression, cells were infected with different doses of viruses (multiplicity of infection [MOI], 0.1 or 10) (Fig. 5D). The results showed that in Marc-145 cells, MALT1 was elevated until 48 h after PRRSV infection at an MOI of 0.1, whereas upon infection at an MOI of 10, MALT1 expression reached its peak at 12 hpi and subsequently decreased. In PAMs, MALT1 was significantly induced after infection at an MOI of 0.1 and then downregulated at 48 hpi. Meanwhile, an MOI of 10 led to a relatively lower expression raise of MALT1 from 12 hpi, suggesting the peak occurs even before 12 hpi in PAMs. These results indicated that MALT1 is dramatically downregulated upon PRRSV infection aggravation after the initial induction of MALT1 upon PRRSV infection.

To confirm the protein degradation pathway of MALT1 after PRRSV heavy infection, proteasome inhibitor MG-132 or lysosome inhibitor chloroquine (CQ) was incubated with PRRSV-infected Marc-145 cells and PAMs. The results showed that treatment with MG-132 recovered MALT1 expression at 48 hpi, whereas CQ made no difference to the downregulation of MALT1 (Fig. 5E). On this basis, coimmunoprecipitation (co-IP) assay was performed to detect the ubiquitination level of MALT1 after PRRSV infection, and the results indicated that MALT1 was ubiquitinated after PRRSV infection compared with uninfected cells, especially at 48 hpi (Fig. 5F), indicating that MALT1 was degraded via the ubiquitin-proteasome pathway after heavy infection of PRRSV.

### nsp6-mediated MALT1 degradation relieves inflammatory responses.

**FIG 6:**
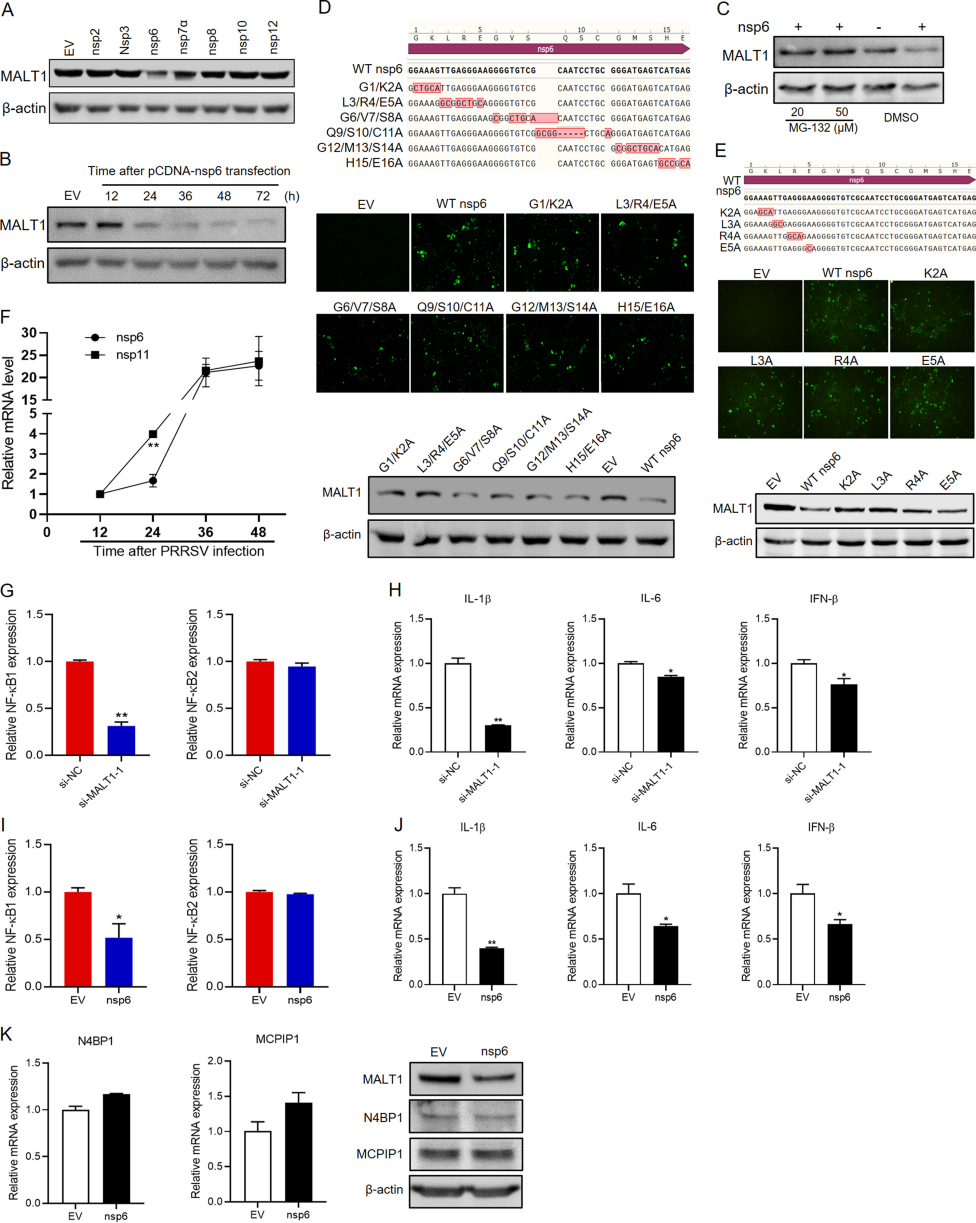
PRRSV nsp6 downregulated MALT1, suppressing inflammatory responses. (A) Plasmids expressing various nsps or empty vector (pCDNA3.1) were used to transfect Marc-145 cells for 24 h. MALT1 protein expression was detected in Western blot analysis. (B) Marc-145 cells were transfected with pCDNA-nsp6. MALT1 levels were determined at the indicated time after transfection. (C) MG-132 was incubated with Marc-145 cells 16 h after nsp6 transfection, and MALT1 levels were detected 24 h after transfection. (D) Sequencing results of nsp6 mutants with two or three amino acids mutated to Ala (top), verification of expression efficiency of various nsp6 mutants in Marc-145 cells in IFA using anti-6×His MAb (middle), and MALT1 protein levels induced by transfection of mutants, along with EV and WT nsp6 (bottom). (E) Sequencing results of nsp6 K2A, L3A, R4A, and E5A mutants (top), verification of the expression efficiency of nsp6 mutants in Marc-145 cells in IFA (middle), and MALT1 expression analysis after transfection with nsp6 or its mutants in Western blot analysis (bottom). (F) mRNA levels of nsp6 and nsp11 in Marc-145 cells at different time points after PRRSV infection. (G and H) Marc-145 cells were transfected with si-MALT1-1 or si-NC for 48 h. mRNA levels of NF-κB1 and NF-κB2 (G) as well as IL-1β, IL-6, and IFN-β (H) were detected in qRT-PCR. (I and J) Marc-145 cells were transfected with pCDNA-nsp6 or empty vectors for 48 h. Expression levels of NF-κB (I) and the indicated cytokines (J) were detected in qRT-PCR. (K) N4BP1 and MCPIP1 mRNA (left) and protein (right) levels after nsp6 transfection.

Furthermore, to identify the vital viral factor that induces MALT1 downregulation, nsps that have potential downregulation effects on MALT1 (Fig. 4C) were used to transfect Marc-145 cells, including nsp2, -3, -6, -7α, -8, -10, and -12. The results of Western blot analysis showed that among these nsps, nsp6 could markedly suppress MALT1 protein expression 24 h after transfection (Fig. 6A). To further confirm the downregulation effect of nsp6 on MALT1, cells were collected at different time points after nsp6 transfection for Western blot detection. MALT1 protein levels were found to significantly reduce from 24 h after nsp6 transfection (Fig. 6B). Moreover, MG-132 treatment in nsp6-transfected cells recovered MALT1 expression (Fig. 6C), indicating the correlation between PRRSV nsp6 and proteasome-mediated MALT1 degradation. Subsequently, to locate the key site of nsp6 responsible for MALT1 downregulation, various mutants with every two or three amino acids in WT nsp6 mutated to Ala were constructed (Fig. 6D), and Marc-145 cells were transfected with these mutants. The results showed that G1/K2A and L3/R4/E5A mutations recovered MALT1 expression. On this basis, single-point mutation of possible active amino acid sites, including Lys 2, Leu 3, Arg 4, and Glu 5, was further performed, and the results of transfection experiments showed that K2A and L3A recovered MALT1 expression (Fig. 6E), indicating that Lys 2 and Leu 3 of nsp6 play critical roles in reducing MALT1 expression. In addition, the transcriptional levels of nsp6 and nsp11 after PRRSV infection were compared. The results showed that nsp11 levels were significantly higher than those of nsp6 at 24 hpi (Fig. 6F), indicating PRRSV manipulates MALT1 expression levels via differential expression of nsps at different stages of infection.

To further explore the effects of nsp6-induced MALT1 downregulation on the innate immune responses, transcription levels of NF-κB and related proinflammatory cytokines, including IL-1β, IL-6, and beta interferon (IFN-β), were detected after nsp6 transfection or MALT1 knockdown by siRNA. The results showed that si-MALT1-1 transfection significantly downregulated transcriptional levels of NF-κB1 (Fig. 6G), along with IL-1β, IL-6, and IFN-β (Fig. 6H), compared with the control siRNA. Consistent with this, nsp6 transfection also inhibited transcriptional levels of NF-κB1 (Fig. 6I) and cytokines (Fig. 6J), indicating that PRRSV nsp6 can suppress host inflammatory responses. In addition, to investigate the impact of nsp6 on RNase expression, gene and protein levels of N4BP1 and MCPIP1 after nsp6 transfection were detected. The results showed that nsp6 transfection had no significant stimulating effects on N4BP1 and MCPIP1 expression (Fig. 6K), leading to no reversion of antiviral activity. Together, these results indicated that PRRSV nsp6 suppressed inflammatory responses but did not recover RNase expression, which builds modulated immune circumstance for virus settlement.

## DISCUSSION

**FIG 7:**
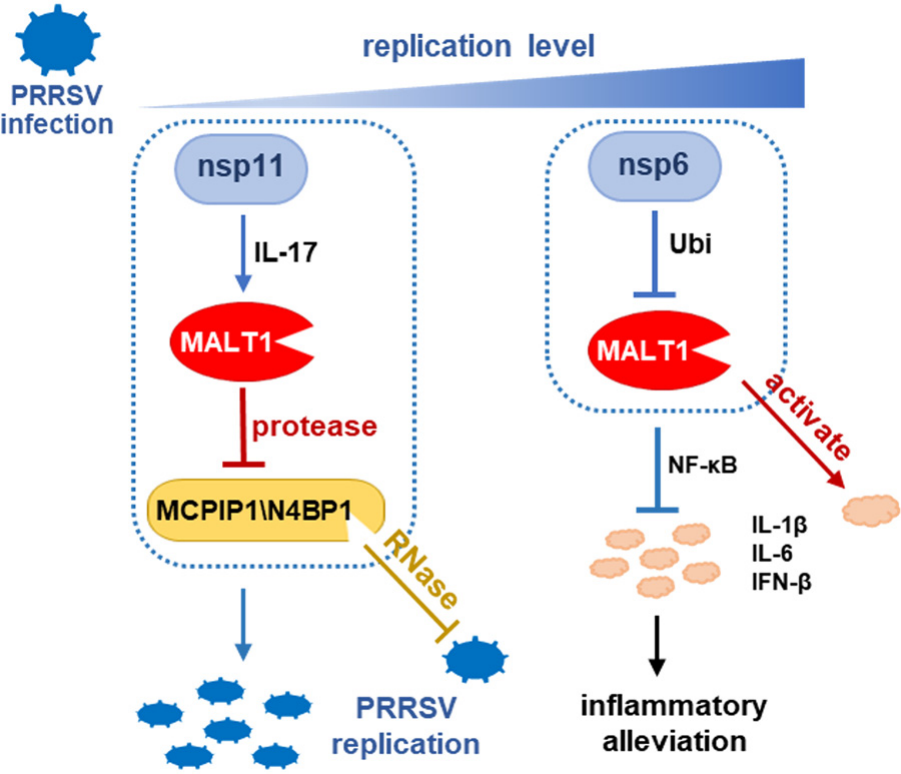
Schematic illustration of PRRSV manipulating MALT1 to adapt antiviral defense for successful infection. Host RNases N4BP1 and MCPIP1 suppress PRRSV replication, relying on their RNase activity. Upon low-level PRRSV infection, PRRSV nsp11 induced MALT1 expression, which degrades antiviral RNases via the proteolytic activity, facilitating PRRSV replication. In contrast, PRRSV nsp6 mediates MALT1 downregulation upon infection aggravation through the ubiquitination-proteasome pathway to alleviate inflammatory responses. Thus, the precise control on MALT1 by PRRSV upon different states of infection contributes to PRRSV infection.

Innate antiviral defense, including host ribonucleases (RNases) and inflammatory responses, serves as the indispensable and immediate barrier in hosts upon infection. Viruses need to develop strategies to evade, subvert, or even hijack these host defense mechanisms to adapt antiviral innate immunity and successfully establish infection. Previous studies have revealed that one of the mechanisms of PRRSV-induced immunosuppression is to inhibit type I interferon expression (4). In this study, PRRSV was confirmed to modulate the innate immunity through another pathway, manipulating MALT1 expression to antagonize host antiviral RNases and alleviate inflammatory responses (Fig. 7). PRRSV has a restricted tropism for monocyte/macrophage lineage in lungs and other tissues and preferentially targets porcine alveolar macrophages (PAMs) (27). Therefore, primarily isolated and cultured PAMs act as an appropriate infection model, which has the comparable soundness of a pig model with minimal animal usage, for pathogenesis research on PRRSV (28, 29) (including this study) and some other major porcine viruses, such as African swine fever virus (ASFV) (30) and porcine circovirus (PCV) (31). PAMs are close to pig tissues *in vivo* and provide a clear platform to reveal the interaction or regulation between host and PRRSV without interference from many other factors in pig infection, such as other pathogen infection and factors from environment (27, 32).

In this study, MCPIP1 and N4BP1 were confirmed to inhibit PRRSV replication, relying on their RNase activity. Both RNases belong to the Zc3h12a-like NYN domain subfamily of endoribonucleases and harbor a similar PIN-like domain with RNase activity (10). MCPIP1 is a spectrum antiviral RNase against various viruses, like HIV-1, by degrading viral mRNA (11). It has been demonstrated that MCPIP1 inhibited PRRSV infection by targeting PRRSV mRNA and inhibiting virus-induced proinflammatory cytokines in our recent study (12). Here, it was further confirmed that Asp125 of porcine MCPIP1 is the key amino acid site for its RNase and anti-PRRSV activity. Notably, a recent study reported that like MCPIP1, N4BP1 also inhibits HIV-1 replication via degrading HIV mRNA species (13). Here, N4BP1 was confirmed to have synergistic effects with MCPIP1 on suppressing PRRSV infection. Interestingly, MCPIP1 is a cytoplasmic RNase, whereas N4BP1 mainly localizes on the nucleus and, thus, might degrade distinguished mRNAs and control different biological processes. Together, N4BP1 cooperates with MCPIP1 to suppress specific virus infection, including HIV-1 and PRRSV. Further studies on the antiviral spectrum of the two RNases and their cooperative interaction should provide additional insights into functions of host RNases.

Although N4BP1 and MCPIP1 directly degrade HIV-1 RNA in quiescent CD4^+^ T cells, MALT1 caused their degradation and inactivation upon T cell receptor stimulation, reactivating HIV-1 proviruses in latently infected cells (11, 13, 14). Thus, MALT1 protease-mediated inactivation of the two RNases, N4BP1 and MCPIP1, facilitates the reactivation of latent HIV-1. Similarly, in this study, MALT1 was confirmed to antagonize expression of N4BP1 and MCPIP1. Importantly, MALT1 was rapidly induced upon PRRSV infection, antagonizing the anti-PRRSV effects of the two RNases relying on its proteolytic activity and, thus, facilitating PRRSV replication. Mi-2 is a small-molecule inhibitor that covalently binds to the catalytic center of MALT1 caspase-like protease domain and inhibits its proteolytic activity (18). In this study, Mi-2 treatment caused accumulation of N4BP1 and MCPIP1 and, importantly, inhibited PRRSV replication. In addition, MALT1 protease activity against the two RNases was further verified by mutation of its caspase-like domain. Porcine MALT1 mutant with C489A was deprived of its proteolytic activity on N4BP1 and MCPIP1 and thus lost its function in promoting PRRSV replication. Together, PRRSV-induced MALT1 expression antagonizes the antiviral effects of host RNases N4BP1 and MCPIP1, relying on its proteolytic activity and, as a result, facilitating PRRSV multiplication. In view of the known and potential antiviral effects of host RNases, the significance of MALT1 protease-induced RNase degradation in pathogenesis of other viruses will be a valuable research direction in the future. In addition, PRRSV nsp4, nsp7β, and nsp11 were confirmed to induce MALT1 expression, especially nsp11. Previous studies verified that several PRRSV nsps, including nsp1α and -1β (33), nsp2 (34), nsp4 (35), and nsp11 (36, 37), induce host immunosuppression via IFN inhibition. Here, PRRSV nsp-induced MALT1 upregulation may be a new mechanism for immune suppression via antagonizing host antiviral RNases. Nsp11 was recently demonstrated to induce IL-17 expression and thus antagonize the anti-PRRSV activity of MCPIP1 (12). In this study, it was further confirmed that IL-17 was responsible for nsp11-induced MALT1 upregulation. Therefore, PRRSV nsp11 may rely on IL-17 to induce MALT1, consequently causing degradation of antiviral RNases like MCPIP1 and N4BP1.

Upon viral infection, various antiviral molecules, such as IFNs and other proinflammatory cytokines, are activated by recognition of pathogen-associated molecular patterns (PAMPs) through a set of pattern recognition receptors (PRRs) (38, 39). Current studies have revealed that PRRSV inhibits the antiviral IFN response via encoding multiple nsps to suppress the RIG-I signaling pathway, degrade MAVS, and interfere with the phosphorylation of IRF3 and the polyubiquitination of IκBα (40–43). Similarly, picornavirus (44), Pox viruses (45), HIV-1 (46), and severe acute respiratory syndrome coronavirus 2 (47) impair IFN response to evade host immune response. Overall, viruses need to relieve innate inflammatory responses to establish infection successfully. Intriguingly, in this study, it was found that infection aggravation of PRRSV induced the downregulation of initially elevated MALT1. MALT1 protein levels dramatically decreased at the late stage of PRRSV infection (especially after 36 hpi) with no strain specificity, and a high dose of viral infection (MOI of 10) induced more rapid decrease of MALT1. Given that MALT1 positively regulates innate immunity and inflammation, the significance of MALT1 decrease is possibly to mollify inflammatory responses induced by viral infection. As expected, in this study, MALT1 knockdown by siRNA inhibited NF-κB signals and production of proinflammatory cytokines, including IL-1β, IL-6, and IFN-β. Specifically, MALT1 facilitates NF-κB activation for proinflammatory gene transcription via both scaffolding and protease activities. On the one hand, as a scaffold protein in the CBM signaling complex, MALT1 recruits and assembles downstream effector proteins for IKK and NF-κB activation (22, 48). On the other hand, the protease activity of MALT1 causes degradation of negative NF-κB regulators for NF-κB signal enforcement, including the NF-κB inhibitor protein A20 (20), deubiquitinase CYLD (19), and NF-κB member RelB (49). Overexpression of MALT1 can lead to nonstop NF-κB activation without the need for upstream signaling (50). MALT1 also cleaves an entire set of mRNA stability regulators for proinflammatory cytokine production, including Roquin-1 and Roquin-2 as well as MCPIP1 and N4BP1 (51). In addition, MALT1 proteolytic activity plays critical roles in JNK (19) and mTOR kinase pathway activation (52). Together, MALT1 acts as an activator in inducing NF-κB activation and proinflammatory cytokine production, while PRRSV-mediated MALT1 downregulation upon infection aggravation avoids aggressive inflammatory responses.

PRRSV drives MALT1 downregulation upon infection aggravation, which is in absence upon stimulation with other PAMPs, like LPS, implying the unique mechanism on MALT1 from PRRSV is involved in the induction of immune suppression. Treatment of proteasome inhibitor MG-132 and co-IP analysis confirmed that MALT1 was degraded via the ubiquitin-proteasome pathway at the late infection stage, and PRRSV nsp6 could independently mediate this process. Further site analysis indicated that Lys 2 and Leu 3 of nsp6 are the critical amino acid sites responsible for MALT1 degradation. Consistent with the results of MALT1 knockdown, nsp6 transfection suppressed transcriptional levels of proinflammatory genes, including IL-1β, IL-6, and IFN-β. These cytokines play key roles in the defense against virus infection. Importantly, nsp6 had no direct effects on expression of anti-PRRSV RNases, including N4BP1 and MCPIP1. Therefore, PRRSV nsp6-mediated MALT1 degradation may be a novel mechanism for PRRSV-induced innate immune suppression. The function of PRRSV nsp6 is largely unknown. One group reported that PRRSV nsp5, nsp6, and nsp7, along with their orthologous nsp6 proteins of several coronaviruses, including infectious bronchitis virus, mouse hepatitis virus, and severe acute respiratory syndrome, can induce small-diameter autophagosomes, activating autophagy (53, 54). Together with the role of nsp6 in proteasome-mediated degradation of MALT1 identified in this study, PRRSV nsp6 seems to act as an activator in the cellular protein degradation system. Together, PRRSV nsps may accurately modulate MALT1 expression at different infection states. PRRSV employs nsps, including nsp4, -7β, and -11, to upregulate MALT1 and antagonize RNase activity, facilitating PRRSV initial replication, and MALT1 was downregulated by PRRSV nsp6 to suppress virus-induced excessive inflammatory responses and rebuild homeostasis in infected host immunity.

In addition, with the discovery of MALT1 proteolytic activity, research and treatment targeting MALT1 proteolytic activity have been reported. MALT1 protease-dead (MALT1 PD) in mice significantly changed the immunophenotype. MALT1 PD mice develop serious autoimmunity with inflammation and lymphocyte infiltration in multiple organs (22, 55), suggesting the role of MALT1 protease activity in immune homeostasis. Notably, regulatory T cell (Treg) numbers are reduced in MALT1 PD mice (22, 56), whereas Th1 and T helper (Th2) cells are increased (55), implying the significance of MALT1 protease activity in T cell activation. Whether MALT1-induced Treg activation contributes to immunosuppression caused by PRRSV deserves further study. In addition, MALT1 protease inhibitor Mi-2 recovers MCPIP1 expression, selectively reducing HIV-1 latently infected CD4^+^ T cells (57). Therefore, MALT1 protease has potential as a therapeutic target against PRRSV infection.

To sum up, PRRSV induced MALT1 protease to antagonize anti-PRRSV effects of the two RNases MCPIP1 and N4BP1, facilitating PRRSV replication. Although multiple PRRSV nsps induced MALT1 expression, nsp6 mediated MALT1 degradation via the ubiquitination-proteasome pathway, indicating elaborate regulatory effects of PRRSV on MALT1 expression. Considering the potential risk in immune pathogenesis caused by excessive MALT1, nsp6-mediated MALT1 degradation may contribute to immune alleviation, which is a benefit for long-term virus infection. For the first time, these findings revealed the precise regulatory machinery of PRRSV on MALT1 for both the antagonistic effects against the host RNases and the recovery of homeostasis in immune system postinfection, enlightening the new mechanism of PRRSV for successful immune suppression and virus survival by manipulating MALT1 expression.

## MATERIALS AND METHODS

### Cell lines and virus.

Marc-145 cells were cultured in Dulbecco’s minimal essential medium (DMEM; HyClone, Thermo Scientific, MA, USA) containing 6% fetal bovine serum (FBS; Invitrogen, USA) at 37°C in 5% CO_2_. 3D4+CD163 cell line was constructed as described previously (12) to help PRRSV replicate in swine macrophages. 3D4/31 and 3D4+CD163 cells were grown in RPMI 1640 (Thermo) supplemented with 10% FBS at 5% CO_2_ and 37°C. Porcine alveolar macrophages (PAMs) were prepared as described previously (58).

PRRSV strain HuN4 (GenBank accession number EF635006.1) was propagated in Marc-145 cells. PRRSV strains ZJnb16-2 (GenBank accession number MH236426.1) and HNxx16 (GenBank accession number MH588709.1 [ORF1a]) were isolated by our laboratory and proliferated in PAMs (6). PRRSV titers were measured by a microtitration assay using Marc-145 cells or PAMs in 96-well plates and calculated as 50% tissue culture infective doses (TCID_50_) per milliliter according to the method of Reed and Muench.

### Virus infection.

Cells were grown to approximately 70% to 80% confluence and infected with viruses. Infection was allowed to proceed at 37°C and 5% CO_2_ for 2 h, and supernatants were then removed. Cell monolayers were rinsed with Hanks’ balanced salt solution to remove unattached virus particles and then incubated in the presence of fresh medium containing 2% FBS at 37°C and 5% CO_2_ for designated time periods to investigate effects of virus infection on target proteins.

### Antibodies and reagents.

Antibodies used in Western blot analysis, immunoprecipitation, immunofluorescence, and immunohistochemistry are the following. MCPIP1 rabbit monoclonal antibody (MAb) (A12667) and N4BP1 rabbit polyclonal antibody (pAb) (A8474) were purchased from ABclonal to detect monkey-derived proteins. Anti-PRRSV N mouse MAb (VH13), anti-porcine MCPIP1 rabbit pAb, and anti-porcine MALT1 mouse pAb were from our laboratory. Anti-N4BP1 rabbit pAb (abs134524) was purchased from Absin to detect porcine N4BP1. Anti-MALT1 rabbit MAb (ab33921) was purchased from Abcam. β-Actin mouse MAb (AF0003) and ubiquitin rabbit pAb (AF0306) were purchased from Beyotime. Anti-Flag tag mouse MAb (D191041) and anti-6×His tag mouse MAb (D191001) were from Sangon Biotech. Goat anti-rabbit IgG (H+L) horseradish peroxidase (HRP)-conjugated secondary antibody (31466) and goat anti-mouse IgG (H+L) HRP-conjugated secondary antibody (G-21040) were from Thermo. Alexa Fluor 555-labeled donkey anti-rabbit IgG (H+L) (A0453) was from Beyotime. Alexa Fluor 488-labeled donkey anti-mouse IgG (H+L) (R37114) was from Invitrogen.

LPS and IL-17 (absin, Shanghai, China), MALT1 inhibitor Mi-2, lysosome inhibitor chloroquine (CQ) and IL-17 inhibitor Y320 (Selleck, China), and proteasome inhibitor MG-132 (Beyotime) were used at the designated concentrations, prepared in dimethyl sulfoxide (DMSO) (Sigma) or water according to the manufacturers’ instructions.

### Expression vector construction and plasmid transfection.

All expression vectors were constructed by homologous recombination using ClonExpress II one-step cloning kit (Vazyme, Nanjing, China). The cDNAs encoding monkey or pig N4BP1 (GenBank accession numbers XM_007992590.2 and XM_003126990.5), MCPIP1 (XM_037997291.1 and XM_021095966.1), and MALT1 (XM_008013951.2 and XM_021100024.1) were obtained from Marc-145 cell or PAM cDNA, respectively, which were subsequently subcloned into the mammalian expression vector pCDNA3.1 or lentivirus vector pCD513B-1. PRRSV nsps were amplified from PRRSV HuN4 strain and cloned into pCDNA3.1 vector. Various mutants of N4BP1, MCPIP1, MALT1, and PRRSV nsp6 were generated from corresponding wild-type constructs. All recombinant plasmids were verified by DNA sequencing. Primers used for plasmid construction are listed in Table 1. Marc-145, 3D4, or 3D4+CD163 cells were transfected with recombinant expression vectors using Lipofectamine 2000 (Thermo) according to manufacturer’s instruction.

**TABLE 1:** Primers for expression plasmid construction

Plasmid	Orienation^*a*^	Primer sequence (5′–3′)
pCDNA-monkey N4BP1	F	TTTAAACTTAAGCTTGGTACCATGGCGGCCCGGGCGGTACTG
	R	TGCTGGATATCTGCAGAATTCTCACTTGTCATCGTCGTCCTTGTAATCATCCAACACCATGGCAGAGAG
pCDNA-pig N4BP1	F	TTTAAACTTAAGCTTGGTACCATGGCGGCCCGGGCGGTGCTG
	R	TGCTGGATATCTGCAGAATTCTCACTTGTCATCGTCGTCCTTGTAATCGTCCAACACCATGGCGGAGAG
pCDNA-pig N4BP1 D624N	F	TTGTTATAAACGGCAGCAATGTTGCAATTACCCATGG
	R	TTGCTGCCGTTTATAACAATGTGCTTCAGATCTGTTC
pCDNA-pig MCPIP1 D125N	F	TGGTCATCAACGGGAGCAACGTGGCCATGAGCCA
	R	TTGCTCCCGTTGATGACCACGGGCCTCAGGTCACCA
pCD513B-monkey MALT1	F	ATAGAAGATTCTAGAATGTCGCTGTTGGGGGACCCGCTGCAG
	R	GATCCTTGCGGCCGCTCATTTTTCAGAAATTCTGAGCCTGTC
pCDNA-pig MALT1	F	GCGTTTAAACTTAAGCTTGGTACCATGCGGCCCGCGGGAGCA
	R	TGCTGGATATCTGCAGAATTCTCACTTTTCAGAAATTCTGAGCCC
pCDNA-pig MALT1 C489A	F	TGGATATGGCGAGGAAAAGAAATGACTATGATGATACC
	R	CTTTTCCTCGCCATATCCAACAGGAACACATTGAGTC

a F, forward; R, reverse.

### RNA interference.

Small interference RNAs (siRNAs) against monkey or pig MALT1 and siRNA-negative control (NC) were designed and synthesized by GenePharma (Shanghai, China). Marc-145 and 3D4 cells were transfected with the indicated siRNAs at a final concentration of 50 nM using Lipofectamine 2000 (Thermo) according to the manufacturer’s instructions for 48 h. Sequences of siRNAs were listed in Table 2.

**TABLE 2:** Sequences of siRNAs

Target gene	siRNA	Sequence (5′–3′)
Control	si-NC	ACGUGACACGUUCGGAGAATT
Monkey MALT1	si-MALT1-1	AUUUGGAGCAUCAACAGGGTT
	si-MALT1-2	AUAAAUGCGUCUGGAGUCCTT
Pig MALT1	si-MALT1-1′	AUUAUUAUUAACUCGGCAGTT
	si-MALT1-2′	AUUUACCCAUAUCUUCUGCTT

### RNA isolation and qRT-PCR.

Total RNA was extracted using the RNA extraction kit (Easy-Do, Zhejiang, China) according to the manufacturer’s instruction and reverse transcribed to cDNA using HiScript III 1st strand cDNA synthesis kit (+gDNA wiper) (Vazyme, Nanjing, China). qRT-PCR was performed using SYBR green PCR mix (Vazyme) on a Stratagene Mx3005P real-time PCR system (Agilent Technologies, Santa Clara, CA, USA). The PCR program included one denaturation cycle at 95°C for 30 s, followed by 40 amplification cycles at 95°C for 5 s and 60°C for 45 s. One final melting cycle to produce a melting curve was added to verify product specificity, and a single peak obtained in the melting curve indicated the specificity of the PCR products. The relative gene expression levels were normalized to the housekeeping gene β-actin or GAPDH. The 2^−ΔΔCt^ method was used to determine the number of fold change in gene expression levels. All primers for qRT-PCR were listed in Table 3.

**TABLE 3:** Primers for quantitative PCR analysis

Target gene	Orienation^*a*^	Primer sequence (5′–3′)
Monkey N4BP1	F	TTTAACTCCTGCCCGGATGG
	R	TGTGTACTGCAGCAGCCTTT
Monkey MCPIP1	F	TGACAAGTTTATGCCTCCCGATGACCC
	R	TGACAAGTTTATGCCTCCCGATGACCC
Pig N4BP1	F	GACTGAGTCAGCCTCTTGGAG
	R	GGGGCTGCATATCTCTAAGGAA
Pig MCPIP1	F	ATGAATCTGTGGGAACTTGAAGACCACAAAGCT
	R	CTACTCATTGAGCTGCTGCGACTTGTAGGAGAGGGAT
PRRSV N	F	CCTCTAGCGACTGAAGATGACGTCAGGCATCACT
	R	ACTCCACAGTGTAACTTATCCTCCCTGAATCT
Monkey β-actin	F	ATCTGGCACCACACCTTCTACAATGAGCTGCG
	R	CGTCATACTCCTGCTTGCTGATCCACATCTG
Pig GAPDH	F	CCTTCCGTGTCCCTACTGCCAAC
	R	GACGCCTGCTTCACCACCTTCT
Monkey MALT1	F	TGCAGGCTTTTATGTCTGTCG
	R	ACGCCATCAACACTTCTCCG
Pig MALT1	F	AGTGGAGTGCACTGAAGATGAAT
	R	CTTGGGGTGCTCCCAGTAAT
PRRSV nsp6	F	GGAAAGTTGAGGGAAG
	R	CTCATGACTCATCCC
PRRSV nsp11	F	CCAAGGTCGCGCATAAC
	R	CATTGTTCTGGGTTGTCAC
IL-1β	F	GGCTTACTACAGTGGCAACG
	R	ATCCAGAGGGCAGAGGTCTA
IL-6	F	TACCCCCAGGAGAAGATTCC
	R	TTTTCTGCCAGTGCCTCTT
IFN-β	F	GCAATTGAATGGAAGGCTTGA
	R	CAGCGTCCTCCTTCTGGAACT
NF-κB1	F	AGCAGATGGCCCATATCTTCA
	R	ATGGGATGGGCCTTCACAAA
NF-κB2	F	CAAGCCCAACTCCGGATCTC
	R	CCCAGAATTTTAGGCGCCCG

a F, forward; R, reverse.

### Co-IP.

Marc-145 cells were lysed using radioimmunoprecipitation assay lysis buffer (RIPA) (Beyotime, Shanghai, China) supplemented with 1 mM phenylmethylsulfonyl fluoride (PMSF) (Beyotime). Immunoprecipitation was performed using protein A+G agarose (Beyotime) according to the manufacturer’s instructions. Briefly, protein samples were first incubated with anti-MALT1 antibodies (1:50 diluted) at 4°C overnight, followed by incubation with 40 μL of protein A+G agarose at 4°C for 2 h. Agarose-Ab-antigen complexes were washed with ice-cold phosphate-buffered saline (PBS) five times and then resuspended with 40 μL 1×SDS-PAGE buffer. Precipitates and whole-cell lysates were subjected to 12% SDS-PAGE, and MALT1 ubiquitination was analyzed by Western blotting using ubiquitin rabbit pAb (1:500; Beyotime).

### Western blotting.

Briefly, cell or tissue samples were lysed using RIPA (Beyotime) to extract total proteins. Protein concentrations were determined using a bicinchoninic acid (BCA) protein assay kit (Beyotime) and 50 μg of protein was separated by 12% SDS-PAGE and then transferred onto polyvinylidene difluoride (PVDF) membranes (Merck Millipore, Billerica, MA). Membranes were blocked with 5% nonfat milk in Tris-buffered saline-Tween 20 (TBST) for 1 h at room temperature. Rinsed blots were then incubated with primary antibody overnight at 4°C, followed by HRP-conjugated goat anti-mouse or anti-rabbit IgG (1:5,000) at 37°C for 1 h. Signals were detected with SuperSignal West Pico/Femto chemiluminescent substrate (Thermo Scientific, MA, USA), and images were captured with a Gel 3100 chemiluminescent imaging system (Sage Creation Science, Beijing, China).

### Indirect immunofluorescence assays and confocal microscopy.

Briefly, cells in culture plates (Costar; Corning, NY, USA) were first fixed with 4% paraformaldehyde for 20 min at room temperature and then permeabilized with 0.2% Triton X-100 in PBS for 15 min. Fixed cells were washed with PBS and blocked with 5% goat serum in PBS for 1 h at 37°C to prevent nonspecific binding. Cells were incubated with corresponding primary antibodies at 37°C for 1 h and stained with Alexa Fluor-conjugated IgG (H+L) (1:1,000) in the dark at 37°C for 1 h. Cellular nuclei were counterstained with nuclear dye 4′,6-diamidino-2-phenylindole (DAPI) (1:2,000 diluted in PBS; Beyotime) for 5 min. Cells were observed under either inverted fluorescence microscope (Olympus Corporation, Tokyo, Japan) or confocal laser scanning microscope IX81-FV1000 (Olympus). Images were generated using FluoView FV1000 software version 3.1.2.2 (Olympus).

### Statistical analysis.

Data were obtained from at least three independent experiments for the quantitative analysis and were expressed as means ± standard errors of the means. All statistical analyses were performed with *t* test or one-way analysis of variance (ANOVA). A *P *value of <0.05 was considered a significant difference.
